# A Modified Entropy-Based Approach for Identifying Gene-Gene Interactions in Case-Control Study

**DOI:** 10.1371/journal.pone.0069321

**Published:** 2013-07-18

**Authors:** Jaeyong Yee, Min-Seok Kwon, Taesung Park, Mira Park

**Affiliations:** 1 Department of Physiology and Biophysics, Eulji University, Daejeon, Korea; 2 Department of Bioinformatics, Seoul National University, Seoul, Korea; 3 Department of Statistics, Seoul National University, Seoul, Korea; 4 Department of Preventive Medicine, Eulji University, Daejeon, Korea; University of North Carolina, United States of America

## Abstract

Gene-gene interactions may play an important role in the genetics of a complex disease. Detection and characterization of gene-gene interactions is a challenging issue that has stimulated the development of various statistical methods to address it. In this study, we introduce a method to measure gene interactions using entropy-based statistics from a contingency table of trait and genotype combinations. We also developed an exploration procedure by using graphs. We propose a standardized relative information gain (RIG) measure to evaluate the interactions between single nucleotide polymorphism (SNP) combinations. To identify the *k* ^th^ order interactions, contingency tables of trait and genotype combinations of *k* SNPs are constructed, with which RIGs are calculated. The RIGs are standardized using the mean and standard deviation from the permuted datasets. SNP combinations yielding high standardized RIG are chosen for gene-gene interactions. Detection of high-order interactions and comparison of interaction strengths between different orders are made possible by using standardized RIG. We have applied the proposed standardized entropy-based method to two types of data sets from a simulation study and a real genetic association study. We have compared our method and the multifactor dimensionality reduction (MDR) method through power analysis of eight different genetic models with varying penetrance rates, number of SNPs, and sample sizes. Our method shows successful identification of genetic associations and gene-gene interactions both in simulation and real genetic data. Simulation results suggest that the proposed entropy-based method is better able to detect high-order interactions and is superior to the MDR method in most cases. The proposed method is well suited for detecting interactions without main effects as well as for models including main effects.

## Introduction

One of the major goals of human genetics is to identify the relationships between genotypes and disease status. Although single-locus approaches have successfully identified many genetic determinants of disease susceptibility, such approaches cannot adequately explain the genetic contribution to complex diseases such as hypertension, diabetes, and certain psychiatric disorders. This phenomenon may be a result of interactions between genetic factors and influences from environmental factors.

Various statistical methods have been proposed for the detection and characterization of gene-gene interactions in case-control studies [1]–[4]. Logistic regression is a traditional parametric approach to the modeling of relationships between genotypes and binary phenotypes. However, for high-order interactions, logistic regressions may produce large standard errors resulting in increased type I errors due to sparse and empty cells [5]. It is also known to have reduced power to detect high-order interactions [6]. Multifactor dimensionality reduction (MDR) is a popular non-parametric approach that characterizes the SNP combinations into “high risk” or “low risk” categories according to the ratio of the numbers of cases and controls. It converts a high-dimensional contingency table to a one-dimensional model without raising the issue of sparse cells [7]. However, this approach could be considered to be overly simplistic and is sensitive to small changes in cell frequencies. Several variants of MDR have been recently developed [8]–[10].

In this article, we focus on the entropy-based approach as an alternative method. Entropy is commonly used in information theory to measure the uncertainty of random variables [11]. There are several approaches that have adopted entropy-based measures to identify the relationships between genes and disease. Bush *et al.* [12] used the normalized mutual information (NMI) method as a measure to evaluate MDR model fitness. Kang *et al.* [13] proposed an entropy-based procedure to detect genetic associations for the case-only design method. Dong *et al.* [14] defined the gain ratio to combine a genetic model with two-locus gene-gene interactions. More recently, Chanda *et al.* [15] proposed an information-theoretic gene-gene and gene-environment interaction analysis of quantitative traits. In this study, we developed a more comprehensive and flexible framework for detecting and interpreting gene-gene interactions. Here we define the standardized relative information gain (RIG) by subtracting the mean values and dividing by the standard deviation of the relative information gain from permuted datasets and apply it to contingency tables of genotype combinations and disease status. It could account for the improper inflation of relative information gain commonly observed with higher order of interactions.

After a brief review of entropy in section 2.1, we have described a new entropy-based procedure for modeling genetic interactions in section 2.2. We have also illustrated the proposed method using two different genotype datasets in sections 3.1 and 3.2. In section 3.3, we have described the simulation study conducted to compare the powers of the proposed method and MDR. Discussions and final conclusions are included in section 4.

## Methods

### Definition of Entropy

The term entropy usually refers to the Shannon entropy, which plays a central role in information theory as a measure of information, choice, and uncertainty contained in a system consisting of a random variable [11]. It quantifies the amount of average information necessary to remove any uncertainty from the system.

If *X* and *Y* are discrete random variables, then the following four entropy values can be computed: 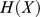, 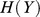, 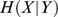 and 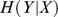. The Shannon’s entropy of *Y* is defined by the following equation:
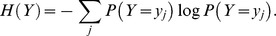



The conditional entropy 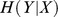 is defined as the average specific conditional entropy of *Y*:
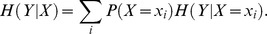
where 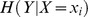 is the entropy of *Y* when 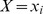. 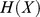 and 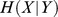 are defined similarly. The information gain (IG) and relative information gain (RIG) are given by



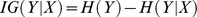
and



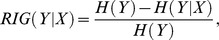
respectively [16]. The RIG value is often called the normalized mutual information (NMI). It quantifies the proportion of information contained in the *X* variable that is transferred to the *Y* variable. It is also interpreted as the amount by which the model reduces the uncertainty about the true state of affairs [12].

### Entropy-based Procedure for Modeling Gene-gene Interactions

The procedure can be summarized in four stages as follows: (i) construction of a contingency table, (ii) calculation of initial relative information gain, (iii) standardization of relative information gain, and (iv) visualization.

#### [Step 1] Construction of a 2-way table

At the first stage, we constructed a 2-way contingency table of the genotypes and disease status. For two-locus interactions in the case-control study, we constructed a 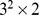 contingency table because there are 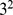 possible genotype combinations and dichotomous disease status. A 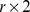 table was constructed for the *k* ^th^ order interactions in the case-control study, where 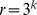 (Table 1).

**Table 1:** Contingency table for *k^th^* order interaction.

SNP					Disease status		total
Combination	SNP1	SNP2	…	SNPk	case	control	
1	AA	BB		KK	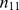	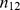	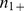
2	Aa	BB		KK	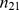	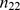	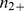
3	aa	BB		KK	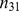	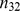	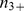
:	:	:	:	:	:	:	:
:	:	:	:	:	:	:	:
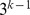	aa	bb		Kk	:	:	:
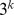	aa	bb		kk	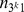	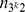	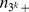
**Total**					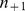	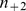	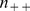

*n_ij_* means the number of samples with *i* ^th^ joint genotype for each SNP combination and *j* ^th^ disease status.

#### [Step 2] Calculation of Initial RIG: 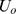


We calculated the initial relative information gain 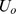 from the constructed contingency table. Let *Y* be the dichotomous disease status and *X* be the SNP combinations; then.
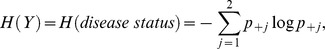


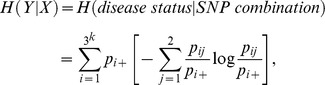
and



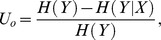
where_
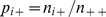
_, 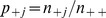 and 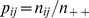.

Note that 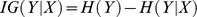 is equivalent to the log-likelihood ratio statistic divided by 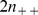 for the independence test of *X* and *Y*. Therefore, 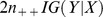 asymptotically follows a chi-square distribution with 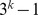 degree of freedom under the null hypothesis of independence. This approximation, however, is not expected to hold for sparse tables in higher order interaction analysis.

We calculated 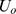 for all the possible combinations of SNPs. A larger 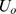 value indicates stronger association between a specific SNP combination and the disease status. It should be noted that empty cells do not cause any ambiguity in the definition. This feature proves advantageous when estimating high-order interactions, because empty cells are common in high-order SNP combinations.

#### [Step 3] Standardization of RIG: *U_r_*

An ensemble of datasets was generated from original data by repeated shuffling of the phenotypes while all genotypes remained fixed. Relative information gains (RIGs) were calculated for each permuted data set by following the same procedures given in Steps 1 and 2. To standardize the RIG, the empirical null distribution of the maximum value of RIG was obtained for each order of interaction. Let 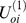 denote the maximum RIG of the *i* ^th^ permuted data set. Then, the average and the standard deviation of 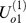,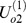,…, 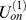 can be computed as follows:
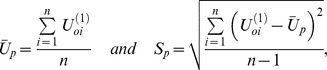
where *n* is the number of the permuted data sets in the ensemble. Standardized relative information gain, 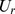, corresponding to initial relative information gain of the original data, 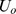, is defined as follows:



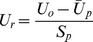



Note that 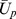 and 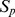 need to be computed for each order of interaction. The empirical null distribution of the maximum value of RIG was used for controlling the family-wise error rate of the multiple comparisons [17], [18]. The adjusted p-values could be obtained by counting the number of 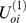 greater than 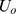.

There is an additional advantage in using 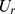 over 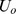. The values of 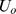 can be shown to increase with the order of interactions, when SNPs in a lower order interaction are a subset of SNPs in a higher order interaction. That is, the values of 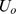 tend to increase regardless of the true additional contribution, as the number of SNPs increases. However, by using 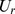, a direct comparison of the association strengths between models with different orders was made possible. Therefore, for the main criteria of the association strength, 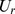 is a more appropriate candidate than 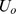 is.

**Figure 1:**
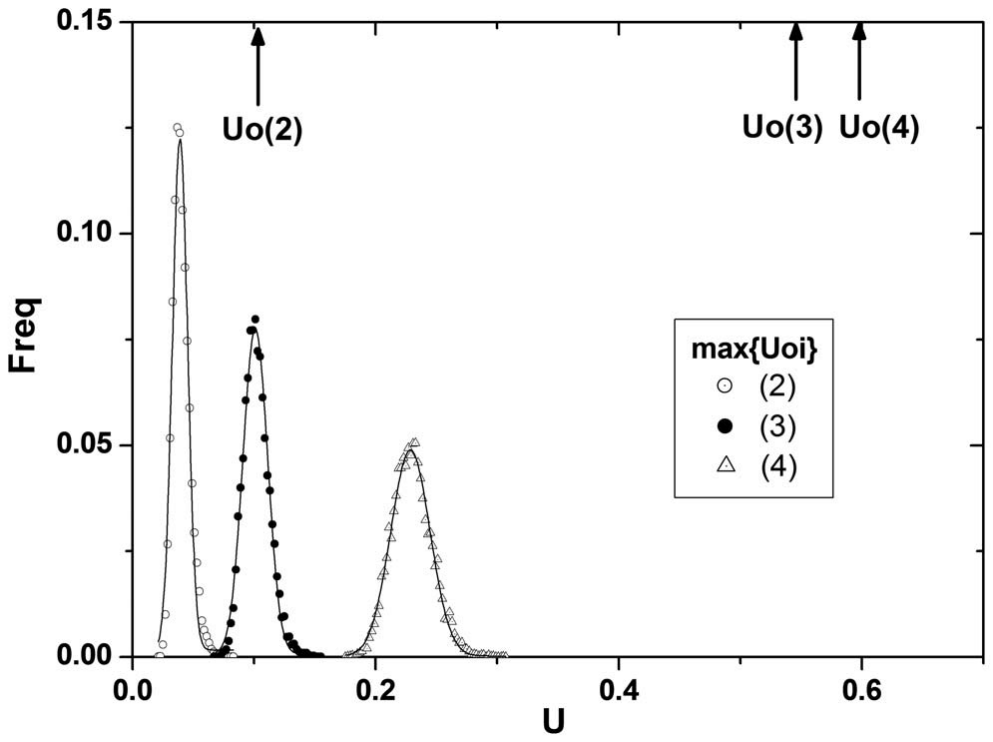
Visualization of the properties of the proposed measures using MDR open-source data. The arrows on the upper side of the graph represent the largest observed 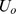 in each order of interactions. The distributions are the null distribution of 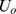 obtained by collecting the maximum 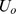
s from each permuted data. Order of interaction is denoted within the parentheses.

Figure 1 shows the properties of the proposed measures. As the order of interaction increased, the largest values of 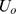 increased, which results from the way in which the mutual information is defined. Note that the empirical distributions for null hypothesis also shifted to the right and became wider as the order increased. Therefore, although the top ranked 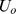 for 3^rd^ order interactions was smaller than that for 4^th^ order interactions, direct comparison of association strength by 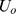 may be biased. In the next section, it will be shown that more reasonable comparison of the association strengths can be made by using the standardized measure . Figure 1 is based on MDR open source data, where the association strengths have unusually large values.

#### [Step 4] Visualization

To identify the SNP combinations with strong interactions at a glance, we use scree plot of 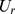. After estimating 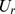 for all the possible *k* ^th^ order SNP combinations, we ranked those values. The scree plot is drawn by plotting 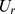
s against their rank; in that way the strength of the interactions may be visualized more clearly. The scree plot is known as an exploratory method to determine the optimal number of factors, and needs neither threshold nor fixed percentage. A typical “above the elbow” approach [19] could be adopted to choose the last substantial drop. The number of points before the last drop was taken as the number of SNP combinations with strong interactions. The line of cut-off value of 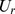 for the given significance level can be added to the plot to identify the significant SNP combinations. For example, the cut-off value of the 5% significance level can be calculated from the upper 5% point of the empirical null distribution of the maximum value of RIG.

We have added one more step for the visualization of two-locus interactions. For *p* SNPs, a 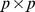 distance matrix was constructed, whose 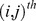 element is 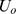 for two-locus interaction between *i* ^th^ and *j* ^th^ SNPs. Multi-dimensional scaling was applied to this matrix. Then, the distance between two SNPs in the graph approximated the strength of two-locus interactions measured by relative information gain. Multi-dimensional scaling (MDS) analysis displays the distance between two SNPs in the graph, which approximates the strength of two-locus interactions measured by the relative information gain. By keeping the point size proportional to the relative information gain from one locus model, 1^st^ and 2^nd^ order interactions could be presented simultaneously. MDS plots can be also constructed by 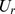
after adding a positive constant such that 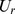 would be non-negative. The resulting MDS plot is equivalent to that produced by 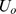.

## Results

In order to demonstrate the proposed entropy-based method, we applied it to two data sets. One is from the MDR open source site (http://www.multifactordimensionalityreduction.org/), and the other is from genetic association study of atopic dermatitis [20]. We generated an ensemble of 1000 permuted data sets with replacement.

### Analysis of Data: Open Source MDR Data

**Figure 2:**
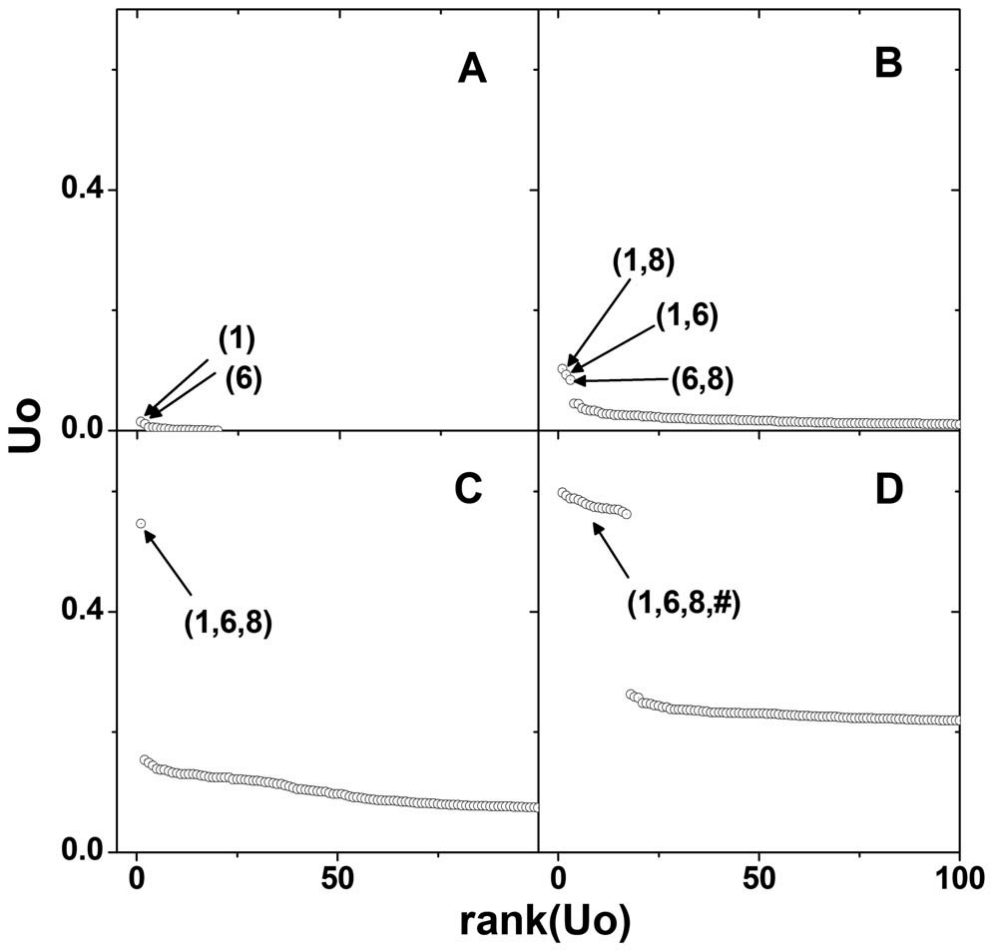
Scree plots of 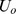 for MDR open-source data. Main effects (A), 2^nd^ order interactions (B), 3^rd^ order interactions (C) and 4^th^ order interactions (D) are shown. The observed relative information gain, 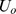, is plotted against the rank determined by the magnitude of 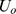. Only the top 100 ranked 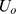
s are plotted for each order of interaction. Top ranked SNP names are denoted within the parentheses.

The open-source MDR data consisted of 20 SNPs and 400 samples. By the MDR method, SNP combinations (1), (1, 8), (1, 6, 8), and (1, 2, 6, 8) were selected for the first to fourth order interactions, respectively. Figure 2 illustrates the initial relative information gain 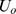. The values of 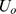 tended to increase with the interaction dimensions. For the 1^st^ order interaction in Figure 1(a), SNP (1) shows the strongest association and followed by SNP (6). For the 2^nd^ order interactions, there are three pairs that seem to be quite different from the others (Figure 2(b)). In the 3^rd^ order interactions, a single SNP combination (1, 6, 8) shows eminent association strength (Figure 2(c)). For the 4^th^ order interactions, all the upper group of SNP combinations included (1, 6, 8) as a subset. These combinations appeared to contain a carryover amount of association strength from that particular 3^rd^ order interaction (Figure 2(d)). The adjusted p-value of 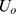 of the best combinations for the first order interaction is 0.292, while the corresponding adjusted p-values for the 2^nd^ to 4^th^ order interactions are less than 0.001.

**Figure 3:**
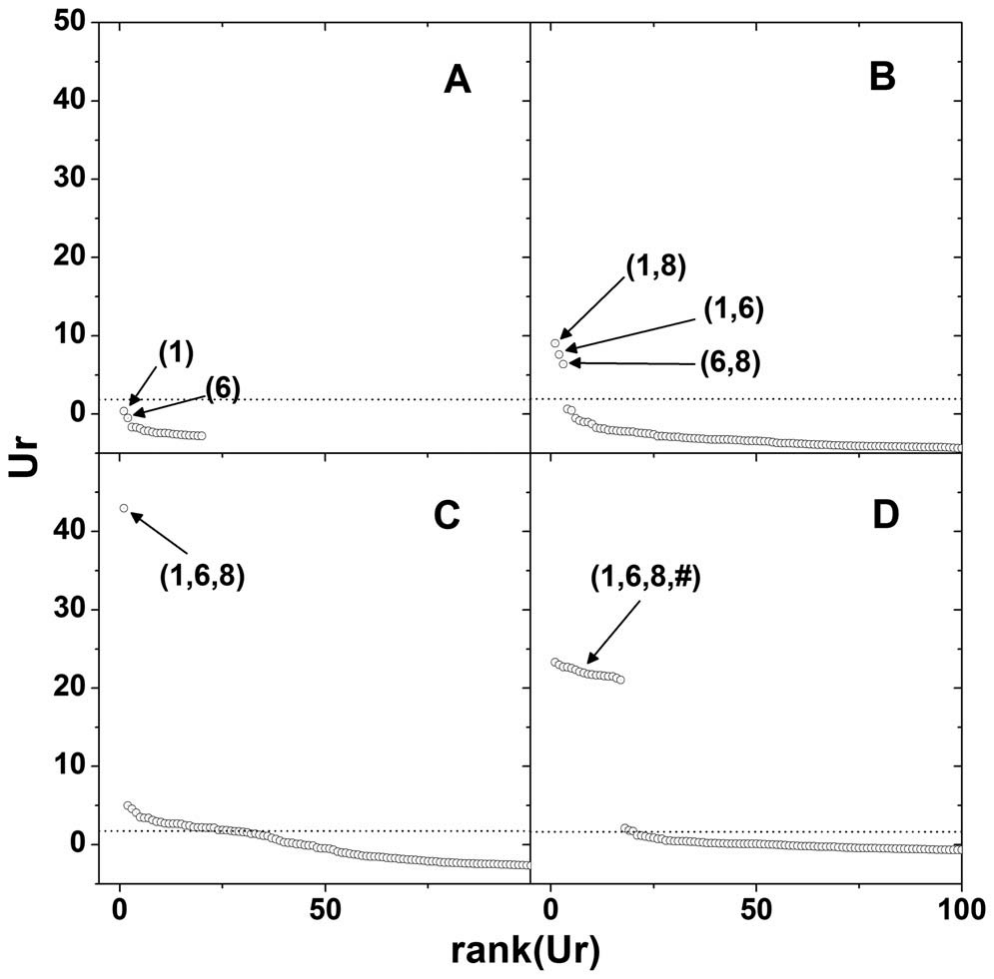
Scree plots of 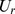 for MDR open-source data. Main effects (A), 2^nd^ order interactions (B), 3^rd^ order interactions (C) and 4^th^ order interactions (D) are shown. The standardized relative information gain, 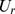, is plotted against the rank determined by the magnitude of 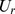. Open-source sample set is used to show the plausibility of using 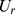. Only the top 100 ranked 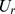
s are plotted for each order of interaction. Top ranked SNP names are denoted in parentheses. The dotted lines show the upper 5% cut-off values of 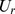 in the empirical null distribution. SNP combinations above the line may be interpreted as significant at 5% significance level.

Figure 3 is the scree plot with the standardized measure, 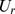. The group of combinations including (1, 6, 8) in the 4^th^ order interactions found to have lower values than (1,6,8) itself after the adjustment, while the (1, 6, 8) in 3^rd^ order interaction maintained its prominence throughout the orders of interactions examined. It was understood that obtaining 4^th^ order interactions by adding any single SNP into the combination of SNPs 1, 6, and 8 actually lowered the association strength from (1, 6, 8). We conclude that a 3-locus interaction involving SNPs identified as 1, 6, and 8 is the most appropriate model. The upper 5% cut-off values for 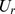 were 1.877, 1.933, 1.761 and 1.634 from the 1^st^ to 4^th^ order interactions, respectively, and were represented by the dotted lines. SNP combinations above the line may be interpreted as significant at the 5% significance level. According to this criterion, only the three combinations pointed by arrows in Figure 3(b) were found to be significant among the 2^nd^ order interactions. In addition to the most promising combination of (1, 6, 8), a few more combinations were significant, as shown in Figure 3(c). All of them were found to share the SNP pairs of (1, 6) or (1, 8) as a subset.

**Figure 4:**
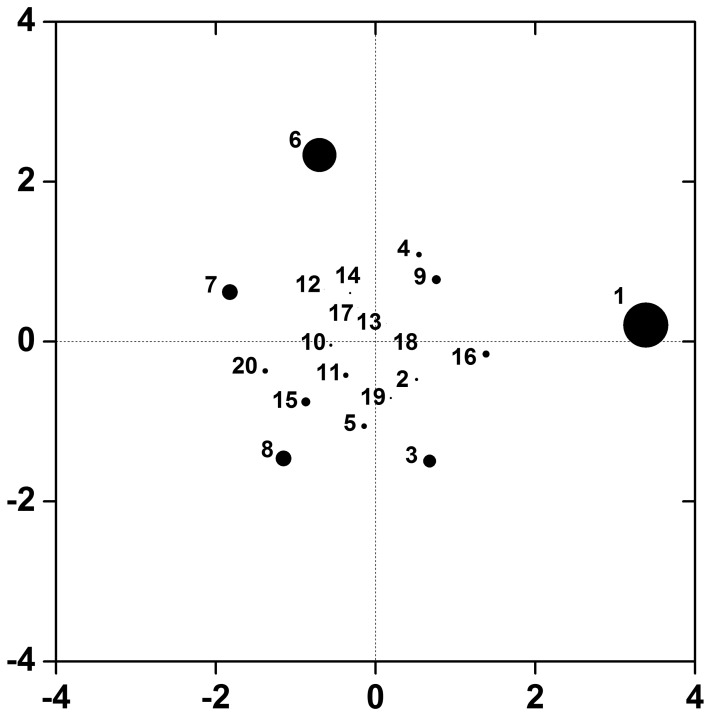
MDS plot for MDR open-source data. Multi-dimensional scaling plot is produced using 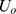 of the 2^nd^ order interactions. The distance between two points approximates the interaction between the corresponding SNPs. The size of the points is proportional to the size of the main effects.

Figure 4 is a multi-dimensional scaling (MDS) plot for 2-locus gene-gene interactions. We used 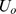 for the construction of the distance matrix. Sizes of the points represent the strength of the main effect of each SNP to the disease. The distances between the two points approximate the degree of gene-gene interactions, although there is loss of information due to dimension reduction via MDS. Point 1 shows the strongest main effect and also the points 6, 7, and 8 show large main effects. The distances between them are prominent among others and represent strong gene-gene interactions.

### Analysis of Real Data: ATOPIC DERMATITIS DATA

This data set was collected from 433 atopic dermatitis patients with allergic type and 474 normal subjects [20]. A total of 17 SNPs were genotyped from 5 genes (IL5, IL8, IL5R, IL8RA, and IL8RB). In this study, 385 cases and 440 controls with complete genotype data were included, as done in the study by Namkung *et al.* [21]. The best combinations chosen by MDR evaluated by balanced accuracy in each dimension are (rs2522411), (rs2522411, rs340808), (rs2290610, rs17882210, rs340808), and (rs17026903, rs340808, rs334809, rs4073), respectively. The corresponding average cross validation consistencies (CVCs) for 10 replications are 7.0, 5.9, 3.5, and 4.5, respectively [21].

**Figure 5:**
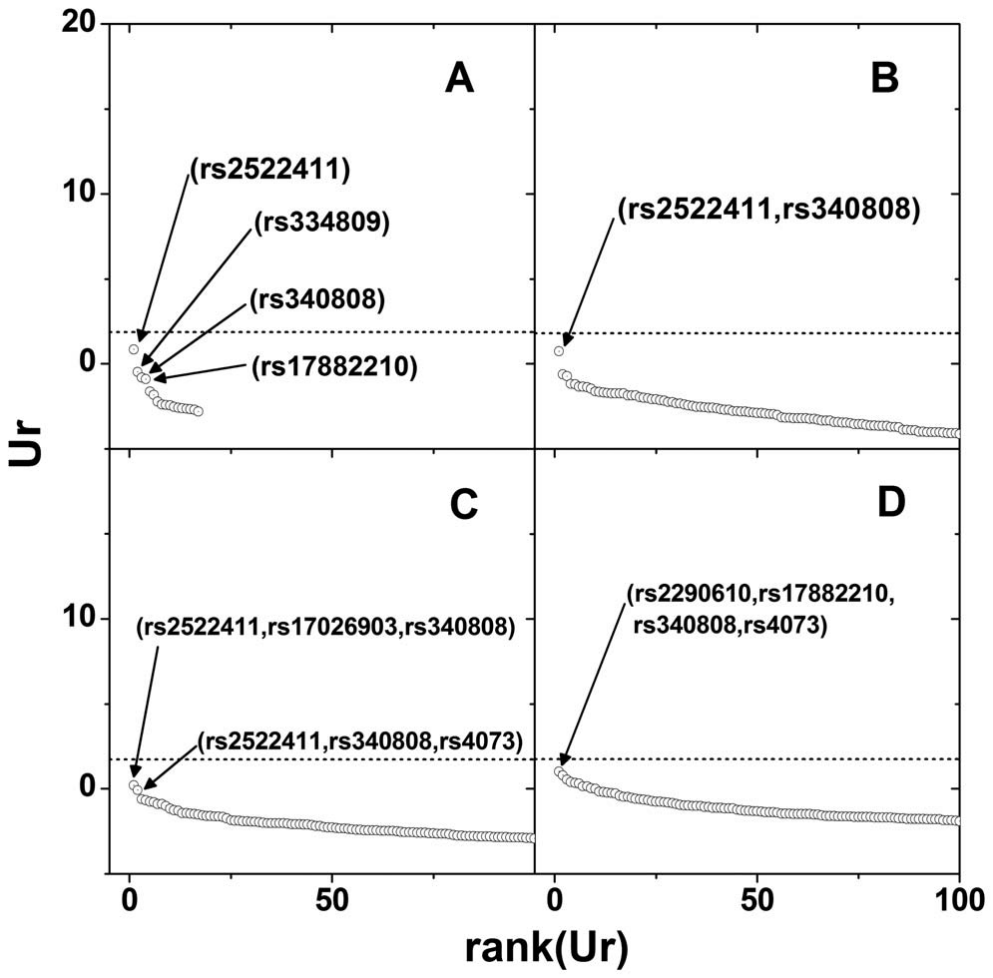
Scree plot of 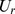 for atopic dermatitis data set. Same plot arrangement as in Figure 3.

Figure 5 is a scree plot of 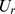. For the 1^st^ order interactions, rs2522411 shows the strongest association with the phenotype and the rs334809, rs340808, and rs17882210 are followed by. For the 2^nd^ order interactions, (rs2522411, rs340808) pair shows the strongest interaction. (rs2522411, rs17026903, rs340808) and (rs2290610, rs17882210, rs340830, rs4073) combinations are the best SNP combinations in the 3^rd^ and 4^th^ order interactions, respectively. Adding rs340808 to the top ranked SNP of rs2522411 in the main effect to convert it into a 2^nd^ order interaction resulted in comparable association strength. On the other hand, adding another SNP, rs17026903 to the pair (rs2522411, rs340808) to make a 3^rd^ order interaction effectively lowered the association strength. This suggested that rs17026903 gave no additional information. For 1^st^ and 2^nd^ order interactions, the best SNP combinations obtained from the proposed method were the same as those obtained from MDR, while the SNP combinations in the best models for 3^rd^ and 4^th^ order interactions were different in the two methods. No significant SNP combinations were detected after adjustment for multiple comparisons. The adjusted p-values of the largest 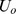 are 0.183, 0.201, 0.388 and 0.162, respectively for the 1^st^ to 4^th^ order interactions. All the points were located under the cut-off line (Figure 5).

### Simulation Results

We evaluated the power of the proposed method in comparison with MDR on the basis of the balanced accuracy (BA) and cross-validation consistency (CVC) for two-locus interactions to assess the performance of the proposed method. We used the same simulation scheme as that of Namkung *et al.* [21]. We took into consideration three different sample sizes (400, 1000, 2000) and five different numbers of SNPs (10, 50, 100, 500, 1000). The numbers of case and control samples were balanced. One pair of SNPs was simulated as a causal factor among all possible combinations. The genotype data of the causal SNPs were generated based on 8 different genetic models (Tables 2, 3). For models 1, 2, and 3, the odds ratio (OR) varied with fixed interaction structure, minor allele frequency, and prevalence. Models 4, 5, and 6 were obtained from Ritchie *et al.* [22], and models 7 and 8 from Bush *et al.* [12]. All the models had little marginal effects. Fifteen different groups of input parameters were used for each model. Total of 100 replicated data sets for each combination of models and groups was used for the power comparison.

**Table 2:** Simulation Scheme based on eight genetic models.

	Model 1			Model 2			Model 3			*Model 4		
MAF/Prevalence	0.1/0.050			0.1/0.050			0.1/0.050			0.1/0.046		
	AA	Aa	aa	AA	Aa	aa	AA	Aa	aa	AA	Aa	aa
BB	**1.21**	0.2	0.2	**1.23**	0.33	0.33	**1.22**	0.4	0.4	0.55	**1.75**	**1.33**
Bb	0.2	**5**	**5**	0.33	**3**	**3**	0.4	**2.5**	**2.5**	**1.54**	0.18	0.74
bb	0.2	**5**	**5**	0.33	**3**	**3**	0.4	**2.5**	**2.5**	**1.75**	0.18	0
	* **Model 5**			* **Model 6**			** **Model 7**			** **Model 8**		
**MAF/Prevalence**	**0.1/0.026**			**0.1/0.017**			**0.2/0.052**			**0.4/0.048**		
	**AA**	**Aa**	**aa**	**AA**	**Aa**	**aa**	**AA**	**Aa**	**aa**	**AA**	**Aa**	**aa**
BB	**1.16**	0.38	0.76	**1.15**	0.40	0.17	0.84	**1.35**	0.80	0.52	**1.07**	**1.89**
Bb	0.38	**3.70**	**1.97**	0.28	**4.23**	**4.89**	**1.30**	0.39	**1.45**	**1.30**	0.92	0.59
bb	0.76	**1.97**	**2.82**	**1.15**	0.06	**5.56**	**1.45**	0.13	**1.04**	**1.21**	**1.08**	0.33

OR is tabulated for each genotype of causal SNPs as in Namkung *et al.* [21]. OR greater than 1 is denoted in boldface; MAF: minor allele frequency;

* from Ritchie *et al.* [22],

** from Bush *et al.* [12].

**Table 3:** Definition of data groups in simulation.

Group		n_SNP				
		10	50	100	500	1000
n_sample	400	1	4	7	10	13
	1000	2	5	8	11	14
	2000	3	6	9	12	15

n_SNP: number of SNPs; n_sample: total number of samples (1∶1 for case:control).

**Figure 6:**
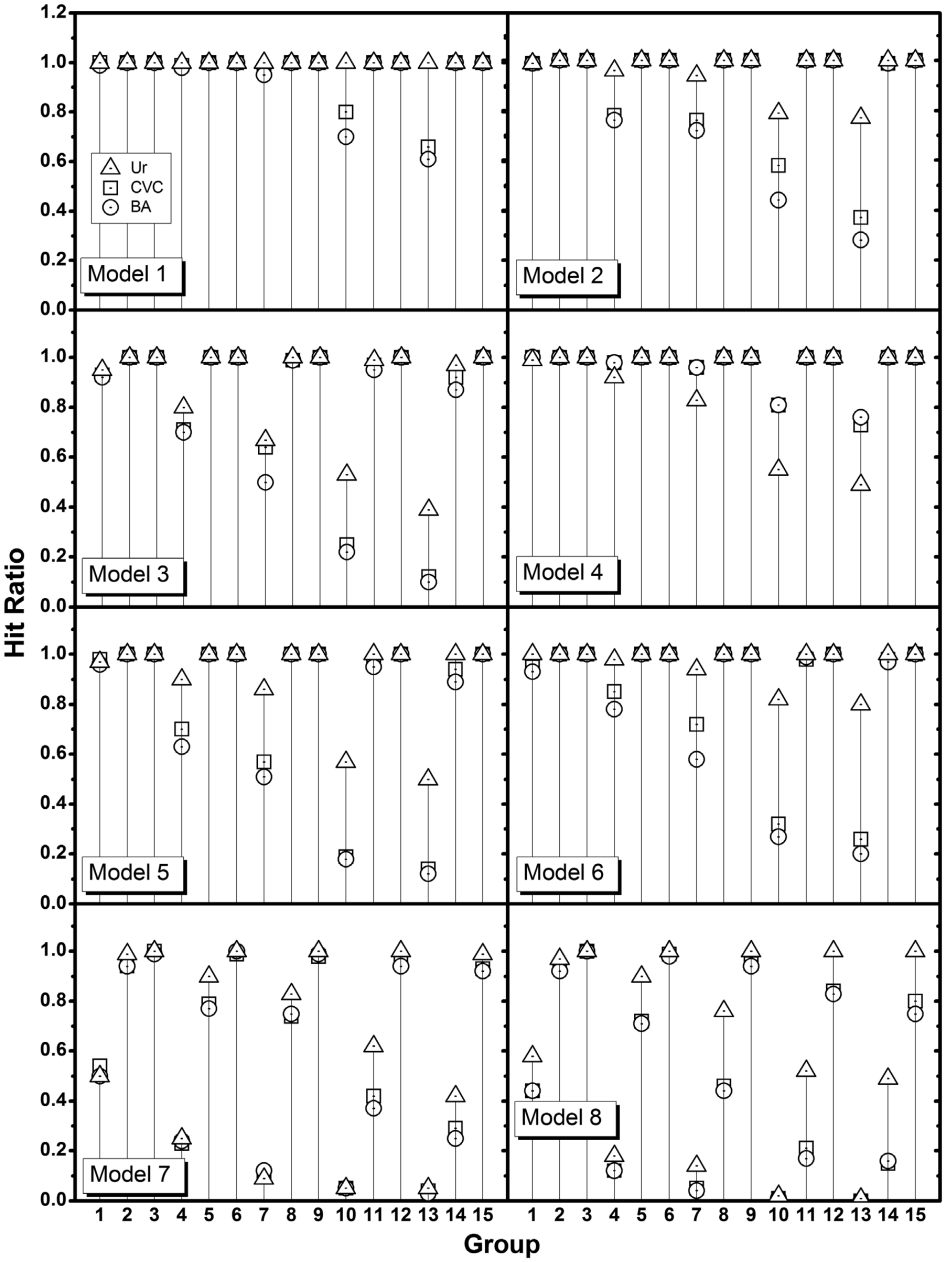
Power comparison between the methods based on entropy and MDR. Hit ratio is used as the empirical power for the fifteen groups each for the eight models. Hit ratio is defined as the ratio at which the incorporated causal pair is identified to have the strongest association. Three different measures, 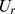, CVC, and BA are compared. Groups 1, 2, and 3 have the same number of SNPs (10), and the numbers of samples increase with the group numbers (400, 1000, 2000), repeating the same for the next 3 groups with an increased number of SNPs (50), and so on. See Table 3 for details. The power of 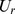 is shown to be higher than the powers of MDR with CVC or BA. The superiority is clearer, especially for the groups 1, 4, 7, 10, 13 in which the number of samples are insufficient when compared to the number of SNPs. As the number of SNPs increases, the difference in power becomes larger.

Empirical power is defined as the proportion of replicated datasets with which the true causal SNP pair is detected as the best pair among all possible two-locus SNP pairs. Each model was run through fifteen groups, varying the number of SNPs and samples. Power of 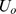 and 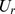 are equivalent because the rank is not changed by standardization. Figure 6 is the graph for empirical power. Two MDR results are clustered together in the plot, although MDR by CVC showed slightly better power than MDR by BA. 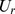 is consistently located well above the CVC, BA groups except for model 4. Groups 1, 2, and 3 have the same numbers of SNPs, and the number of samples increases with the group numbers. The same patterns of number of samples are repeated for the next three groups with an increased number of SNPs, and so on (Table 3). Therefore, there are five subgroups showing similar patterns in these plots. In general, using 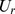 as a measure to find the causal pair seems to be superior to using MDR with CVC or BA. The superiority is clearer, especially for the groups 1, 4, 7, 10, and 13, in which the number of samples is insufficient when compared to the number of SNPs. As the number of SNPs increased, the power difference became larger, which would be a great advantage when dealing with a real data set in which the number of samples is usually far less than the number of SNPs.

## Discussion

In this study, we proposed an entropy-based method, which could identify the high-order gene-gene interactions efficiently. The proposed method utilizes the relative information gain and its standardized measure. Scree plots of the measures enabled us to identify the significant SNP combinations. Direct comparison of the association strengths was possible between different orders of locus interactions. An MDS plot represented 2^nd^ order interactions while representing the degree of the main effects simultaneously. One could calculate the empirical p-values for 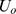 from the permuted datasets in Step 3 of the proposed method.

The proposed method and the MDR with different evaluation criteria were compared by simulation. In this simulation, we focused on the 2^nd^ order interactions. The power obtained varied across the different measures as well as across the genetic models that describe the effect size and the patterns of interactions. The proposed method shows consistent superiority to MDR throughout the examined simulation models. This pattern became clearer in the groups with insufficient numbers of samples when compared to the given numbers of the SNPs.

Computing time of the proposed method would depend directly on the size of the ensemble of the permuted data sets. With 1000 permutation, the computational time from the first to fourth order interactions of the open source MDR data was 1.5 minute using Intel 2.33GHz Quad Core CPU. Atopic dermatitis data took only 1 minute.

The proposed method has been devised mainly for the candidate gene sets. Thus, applying it directly to the genome wide association studies (GWAS) would be infeasible in its current form. For the joint identification of SNPs for GWAS, Cho *et al.* [23] proposed using a pre-screening step and then applying a joint identification step. Our proposed method can be easily applied to the GWAS data after a pre-screening step. For example, when top 1000 SNPs are pre-screened with 2000 samples, our method would take about 1.3 hour for two locus interactions with 1000 permutation. Since the proposed method uses only the mean and variance from the permuted data sets, a large number of permutations is not required. We are investigating more efficient computational approaches for GWAS data such as an adaptive permutation approach [24] which repeats the permutations up until both mean and standard deviation of empirical null distribution are converged. An alternative approach using approximation to the known distribution is also under investigation. The entropy method for GWAS data will be reported separately.

In summary, there are several advantages of the proposed method. It is a non-parametric method and does not assume any prior distribution or any particular genetic model. It provides a list of ranked interactions on the basis of their information gain. It demonstrated better power in most simulation settings. It could perform well with the data that had sparse cells. The performance of our method in the case of sparse data would be considered as a future study.

However, there are some limitations of our proposed method, which can be summarized as follows. First, it is basically an exhaustive search technique as is MDR, and therefore is more suitable for candidate gene studies to find higher-order interactions. Second, it does not separate the main effects from pure interaction effects. Consequently, the SNPs with strong marginal effects but little interaction effects may not be discriminated, although the simulation results suggest that the proposed method is well suited for detecting interactions without main effects.
